# Impact of Hashimoto’s thyroiditis on the tumor microenvironment in papillary thyroid cancer: insights from single-cell analysis

**DOI:** 10.3389/fendo.2024.1339473

**Published:** 2024-09-16

**Authors:** Hongzhe Ma, Guoqi Li, Diwei Huo, Yangguang Su, Qing Jin, Yangxu Lu, Yanyan Sun, Denan Zhang, Xiujie Chen

**Affiliations:** ^1^ Department of Pharmacogenomics, College of Bioinformatics and Science Technology, Harbin Medical University, Harbin, China; ^2^ Department of Urology Surgery, The Fourth Affiliated Hospital of Harbin Medical University, Harbin, China; ^3^ Department of General Surgery, The Fourth Affiliated Hospital of Harbin Medical University, Harbin, China

**Keywords:** single-cell analysis, thyroid-stimulating hormone, immune cell communication, cancer genomics, TCGA-THCA

## Abstract

This study investigates the impact of Hashimoto’s thyroiditis (HT), an autoimmune disorder, on the papillary thyroid cancer (PTC) microenvironment using a dataset of 140,456 cells from 11 patients. By comparing PTC cases with and without HT, we identify HT-specific cell populations (HASCs) and their role in creating a TSH-suppressive environment via mTE3, nTE0, and nTE2 thyroid cells. These cells facilitate intricate immune–stromal communication through the MIF–(CD74+CXCR4) axis, emphasizing immune regulation in the TSH context. In the realm of personalized medicine, our HASC-focused analysis within the TCGA-THCA dataset validates the utility of HASC profiling for guiding tailored therapies. Moreover, we introduce a novel, objective method to determine K-means clustering coefficients in copy number variation inference from bulk RNA-seq data, mitigating the arbitrariness in conventional coefficient selection. Collectively, our research presents a detailed single-cell atlas illustrating HT–PTC interactions, deepening our understanding of HT’s modulatory effects on PTC microenvironments. It contributes to our understanding of autoimmunity–carcinogenesis dynamics and charts a course for discovering new therapeutic targets in PTC, advancing cancer genomics and immunotherapy research.

## 1 Introduction

As the most common endocrine malignancy in the world, the incidence of thyroid cancer has been increasing over the last three decades (1). The global incidence rate in women is three times higher than in men, and the global cancer burden in women is 5.1% (2). Among them, papillary thyroid cancer (PTC) is the most common subtype of thyroid cancer (accounting for 70%~85.9%) (3). Although PTC progresses slowly, a significant proportion of patients have metastases by the time of diagnosis. In the case of metastasis, the combination of surgery, radioactive iodine (RAI) ablation, and thyroid-stimulating hormone (TSH) suppression can still get a favorable prognosis for most cases. However, there are still some metastatic cases that do not benefit from the above treatment strategies (4, 5). Based on traditional genome and transcriptome sequencing techniques, several diagnostic and progressive genes of PTC, such as BRAF and RAS, have been discovered (6). However, this approach ignores the high heterogeneity of PTC. Different tumor microenvironments around PTC will affect the occurrence, development, and drug resistance of tumors. Several studies have reported the effect of tumor-infiltrating immune cells on prognosis in patients with thyroid cancer (7, 8). Myeloid cells increase in proportion in cancer patients and reduce survival time through immunosuppressive function (9). Tumor-associated macrophages vary in frequency in different subtypes of thyroid cancer (10). Natural killer (NK) cells also play a central role in the immune surveillance of thyroid cancer (11). Lymphocyte density was associated with the overall survival and recurrence rate of PTC (12). These immune cells play their respective roles from different aspects. Therefore, systematic evaluation of the tumor immune microenvironment of PTC is helpful to understand the pathogenesis of cancer and guide clinical rational treatment. Hashimoto’s thyroiditis (HT), also known as chronic lymphocytic thyroiditis (CLT), is a common autoimmune endocrine disease, causing hypothyroidism or hyperthyroidism, and the incidence is also increasing year by year. Approximately 18.9% to 23.2% of PTC patients have been reported to have HT, and PTC patients with HT have a better prognosis than PTC patients without HT. However, at the same time, HT is considered to be a chronic inflammatory response, and various inflammatory cells infiltrating around the thyroid of patients with HT can damage the DNA of interstitial cells, leading to erroneous DNA repair, thereby promoting the occurrence of PTC. When HT and PTC occur at the same time, experts at home and abroad have different opinions on whether the former has a protective or promoting effect on the latter (13–17). This indicates that the role of HT in the formation of the tumor immune microenvironment of PTC is still unclear. Therefore, this research will focus on HT development to promote or inhibit PTC.

In the past, the inferCNV algorithm was usually used to distinguish malignant epithelial cells from non-malignant epithelial cells, which is an effective method and widely used. However, the existing problem is how to screen the results obtained by the inferCNV algorithm. The usual selection of the clustering coefficient K with copy number variation is subjective, which will lead to inaccurate results. To solve this problem, we proposed a method to determine the best clustering coefficient K based on TCGA data, which can effectively solve the problem of subjectivity in coefficient selection and provide a new strategy for the clustering coefficient selection of the inference results of single-cell copy number variation in the future.

With the development of single-cell RNA sequencing (scRNA-seq), solving tumor heterogeneity from the perspective of cells has become a hot spot at the forefront. Several studies have reported the use of scRNA-seq in thyroid cancer, such as a recent study on gender differences in the tumor microenvironment in PTC patients (18), the progression of follicular thyroid cancer and medullary thyroid cancer (19, 20), and the dedifferentiation of anaplastic thyroid cancer and PTC (21). To explore whether HT promotes or inhibits the generation and development of PTC, we conducted a comprehensive analysis of the paratumors, primary tumors, lymph nodes, and distant metastasis sites of 11 PTC patients and systematically compared the differences in tumor microenvironments of PTC patients with and without HT. The developmental trajectories of malignant thyroid epithelial cells (mTEs) and non-malignant thyroid epithelial cells (nTEs) and their interaction with HASCs were indicated. This discovery can help us better understand how HT inhibits the development of PTC by affecting its tumor microenvironment. To expand the role of HASCs, we found the relationship between HASCs and prognosis at the single-cell level, and clinical features in TCGA-THCA were further investigated to find the value of HASCs in clinical application. Based on the HASC subtypes, studies have identified unique genomic and drug sensitivity profiles of different molecular subtypes, and this provides a new idea for the personalized treatment of PTC.

## 2 Methods

### 2.1 scRNA-seq data processing

We obtained the number GSE184362 from the Gene Expression Omnibus (GEO) database (22) (https://www.ncbi.nlm.nih.gov/geo/), and a total of 23 samples (6 paratumors, 7 primary tumors, and 10 metastatic tumors), which consisted of 8 samples with HT and 15 samples without HT, were used for analysis via the Seurat R package (23). For each sample, genes were retained with detected expression in more than three cells. Cells with less than 200 detected genes were excluded. Finally, 171,524 cells were preserved. Before correcting batch effects, we used the NormalizeData() function in Seurat to normalize the raw gene expression value by the global-scaling normalization method “Log-Normalize”:


$$Exp_{Normalized=}log^{\frac{Exp_{Raw}}{Exp_{Total}}*10000+1}$$

where Exp(Normalized), Exp(Raw), and Exp(Total) stand for raw gene expression value, normalized gene expression, and the total expression of all genes in one cell, respectively. Then, the “vst” method of the FindVariableFeatures function was used to find the highly variable genes (top 5,000) in each sample. In the process of batch effect correction, we went through three steps. First of all, the SelectIntegrationFeatures function was used to select the integrated dataset required features, and then, the FindIntegrationAnchors function was used to find each anchor point between two datasets. In the end, the IntegrateData function completes the merge of the dataset according to the anchor points identified in the previous step. After batch effect correction, there were 14,0456 cells left over here, and we selected the top 5,000 highly variable genes through the FindVariableFeatures() function, and the top 20 principal components (PCs) were selected based on the JackStraw() function. According to the top 20 PCs, the FindNeighbors() and FindClusters() functions were applied to cluster the cells. The cluster identified 14 cell clusters at a resolution of 0.1, which were annotated into six cell types by marker genes of myeloid cells (LYZ, FCER1G, LYZ, TYROBP), T/NK cells (CD3D, CD3E, IL7R, IL32, TRAC), B cells (CD79A, CD79B, MS4A1, IGKC, CD74), thyroid epithelial cells (TG, CLU, FN1, MGST1, S100A13), fibroblasts (RGS5, IGFBP7, TAGLN, COL1A2, ACTA2), and endothelial cells (TIMP3, RAMP2, CLDN5, TFPI, MGP) (24).

### 2.2 CNV analysis of epithelial cells

To distinguish malignant thyroid epithelial cells (mTEs) from non-malignant thyroid epithelial cells (nTEs), we used the inferCNV R package to predict the copy-number alterations (CNAs) of cells and compared them to the reference “normal” cells (this refers to paratumor cells) from scRNA-seq data (25). By setting the cutoff parameter of the inferCNV package’s run function to 0.1, the HMM_type parameter to i6, and the HMM_report_by parameter to cell, we get the CNA score for each cell. According to the CNA scores of cells on 22 chromosomes, all cells (including paratumors, primary tumors, and metastatic tumors) were clustered using K-means clustering, and the number of clustering K values ranged from 6 to 15.

### 2.3 Developmental trajectory inference of mTEs and nTEs

The Monocle2 R package was used to perform the trajectory analysis for mTEs and nTEs (26). Function newCellDataSet() converted the Seurat object to CellDataSet object, and function estimateSizeFactors() and function estimateDispersions() were used to standardize and normalize the gene expression data of cells, respectively. The genes with average log2 fold change greater than 0.5 and adjusted *P*-values less than 0.05 between HT and non-HT of T/NK cells were used as ordering genes in the trajectory analysis. The DDRTree method of the reduceDimension function was used for dimension reduction. Furthermore, the differentially expressed genes (DEGs) (average log2 fold change >1, adjusted *P*-value<0.05, and *q*-value<0.01) that changed along with the pseudotime were identified by the differentialGeneTest() function. The BEAM function was used to find genes that are regulated in a branching way.

### 2.4 Cell–cell interaction analysis of HASCs

Here, we defined the subset of cells that had a significantly higher percentage of content in PTC samples with HT than in PTC samples without HT as HT-associated specific cells (HASCs). At the same time, the significant difference of this cell subset should be *P<*0.05, while HASCs are more capable of exhibiting differences in the tumor microenvironment between PTC samples with and without HT. Cell–cell communications among HASCs were mapped using the CellChat R package, a common repository of ligands, receptors, cofactors, and their interactions (27). For cell interaction analysis, expression levels were calculated relative to the total read map of the same set of coding genes in all transcriptomes. Expression values were averaged across each single-cell cluster/cell sample.

### 2.5 Identification of molecular subtypes based on HASCs in TCGA-THCA

Transcriptome data from The Cancer Genome Atlas (TCGA) of THCA were downloaded from UCSC XENA (28) (https://xena.ucsc.edu/). Consensus clustering is a method that provides quantitative evidence for determining the number and membership of possible clusters in a dataset. This approach has been widely used in cancer genomics, where new disease molecular subtypes have been discovered. To discover various molecular patterns based on HASCs, the ConsensusClusterPlus R package was employed (29).

### 2.6 The characteristics of molecular subtypes

The ESTIMATE algorithm, which comes true with the IOBR R package, was applied to evaluate the immune score and stroma score of the samples for validation of the molecular subtype signatures found (30, 31). Between the molecular subtypes, the variation in the distribution of genes was depicted by the maftools R package (32). At the same time, the drug sensitivity (IC50 value) of 138 GDSC database drugs was predicted by the pRRophetic R package (33).

### 2.7 Statistical analysis

All statistical analyses were performed using the R tool (v.4.1.1). The Wilcoxon test was applied to compare the differences between two groups, and the Kruskal–Wallis test was used to compare differences between multiple groups of samples. Here, ns indicates *P >*0.05, * indicates *P<*0.05, ** indicates *P<*0.01, *** indicates *P<*0.001, and **** indicates *P<*0.0001. Among them, *P<*0.05 indicates a significant difference. The Kaplan–Meier survival analysis was carried out using the R packages survival and survminer.

### 2.8 Workflow of the experimental design and analysis

**Figure 1:**
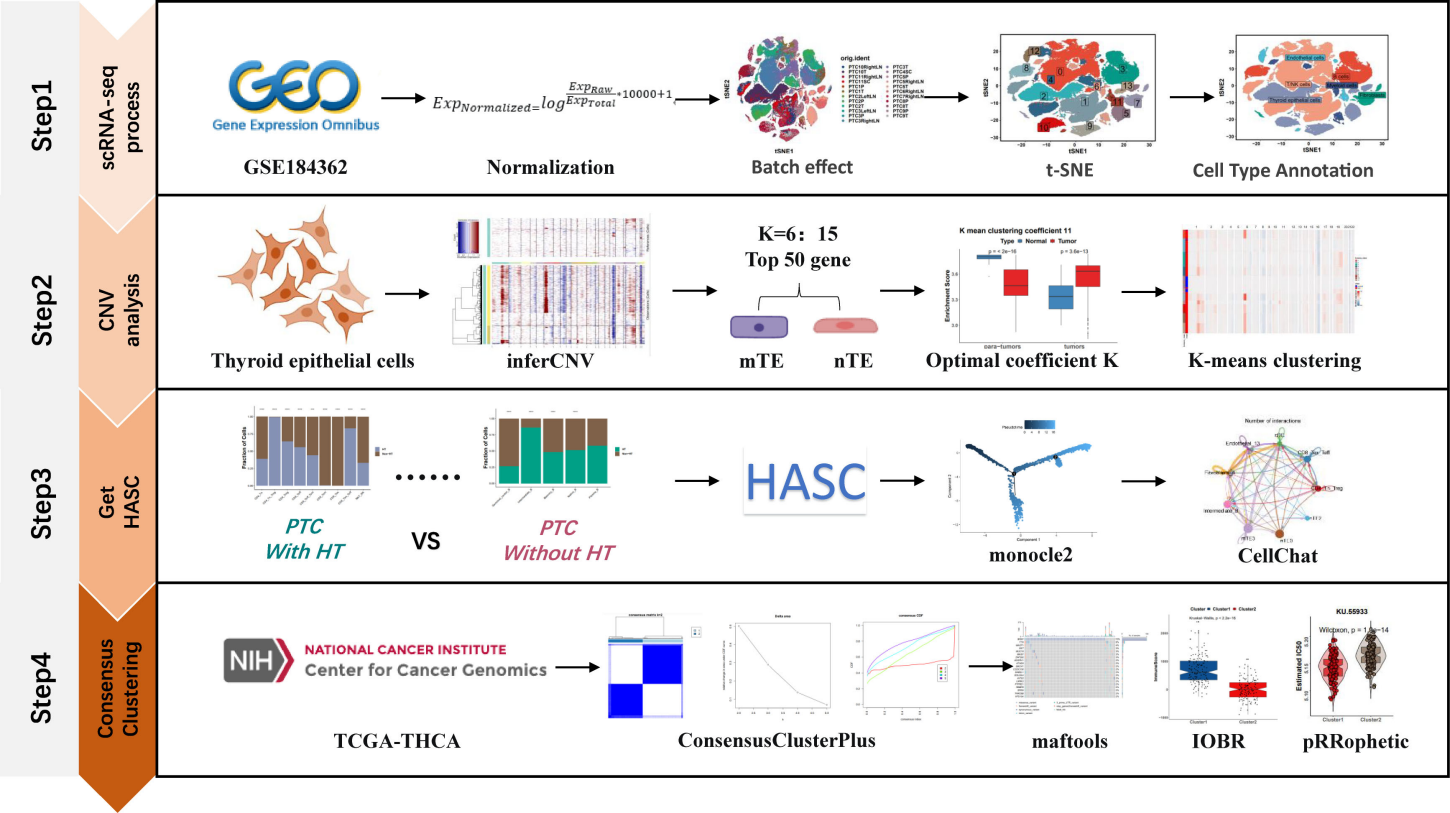
Workflow of the effect of HT on the PTC tumor microenvironment at the single-cell level.

The workflow of this study was divided into four steps as follows (**Figure 1**): the first step is the processing of single-cell data, the second part is the copy-number variation analysis based on the inferCNV algorithm to distinguish malignant and non-malignant epithelial cells, the third step is the acquisition of HASCs, and the fourth step is the molecular typing of TCGA-THCA samples based on HASCs.

## 3 Results

### 3.1 Landscape of PTC by scRNA-seq

**Figure 2:**
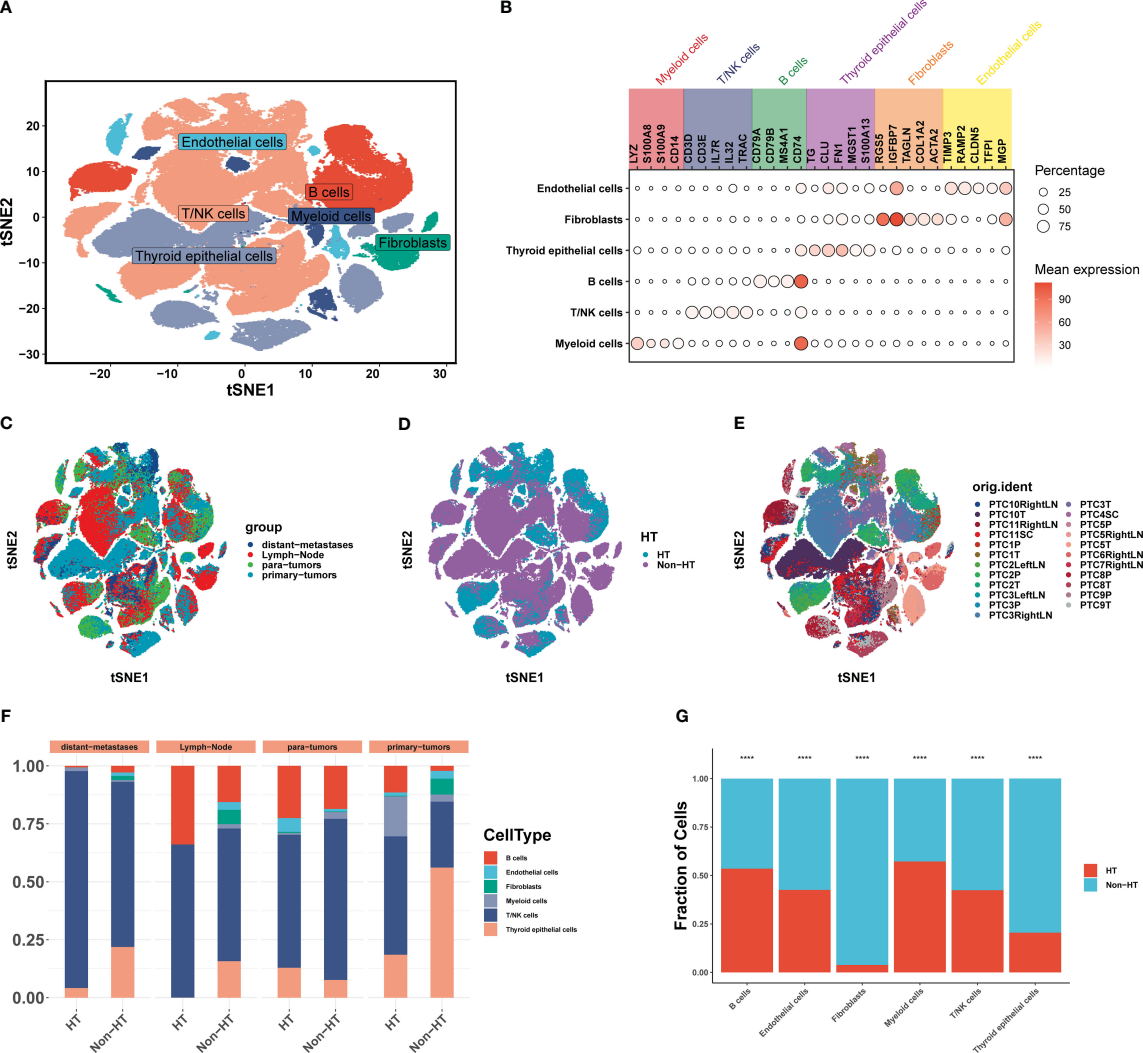
Overview of scRNA-seq analysis across 11 PTC patients. **(A)** t-SNE plot visualizing 14 distinct clusters encompassing 140,456 cells from all samples (*n* = 23), colored by cell type (*n* = 6). **(B)** Bubble plots showing the expression levels of marker genes for each cell type. **(C)** t-SNE plot visualizing cell clusters colored by tissue of origin. **(D)** t-SNE plot visualizing cell clusters colored by HT and non-HT. **(E)** t-SNE plot visualizing cell clusters colored by samples. **(F)** Horizontal bar charts showing the relative abundance of various cell types between HT and non-HT in each tissue of origin. **(G)** *Post-hoc* analysis of each cell between HT and non-HT; *P< 0.05, **P< 0.01, ***P< 0.001, ****P< 0.0001.

After rigorous quality screening, a total of 140,456 cells were retained for further analysis (**Figure 2A**). Six cell types, namely, endothelial cells (TIMP3, RAMP2, CLDN5, TFPI, MGP), fibroblasts (RGS5, IGFBP7, TAGLN, COL1A2, ACTA2), thyroid epithelial cells (TG, CLU, FN1, MGST1, S100A13), B cells (CD79A, CD79B, MS4A1, IGKC, CD74), T/NK cells (CD3D, CD3E, IL7R, IL32, TRAC), and myeloid cells (LYZ, FCER1G, LYZ, TYROBP), were obtained by using t-SNE dimension reduction clustering at low resolution (**Figure 2B**). All these cell subtypes were shared among tissue sources (**Figure 2C**), whether with or without HT (**Figure 2D**), and among samples (**Figure 2E**). It has a mixed biological origin and was not affected by data preprocessing. Overall, compared with non-HT patients, the immune system was significantly activated in HT patients, with more T/NK cells, B cells, and myeloid cells at the cancer site and fewer fibroblast cells. HT, as an autoimmune disease, leads to excessive activation of the immune system, which may inhibit the development of PTC by alleviating the immunosuppressive effect of tumors (**Figure 2F**). The chi-square test revealed significant differences in the content of the six types of cells between HT and non-HT (**Figure 2G**). Among them, the content of immune cells was higher in patients with HT although T/NK cells were excluded, which may be due to the low content of T/NK cells in the HT samples of paratumor tissues. Either way, it is clear that the immune systems of the HT samples were better activated.

### 3.2 Distinguishing between malignant and non-malignant thyroid epithelial cells

**Figure 3:**
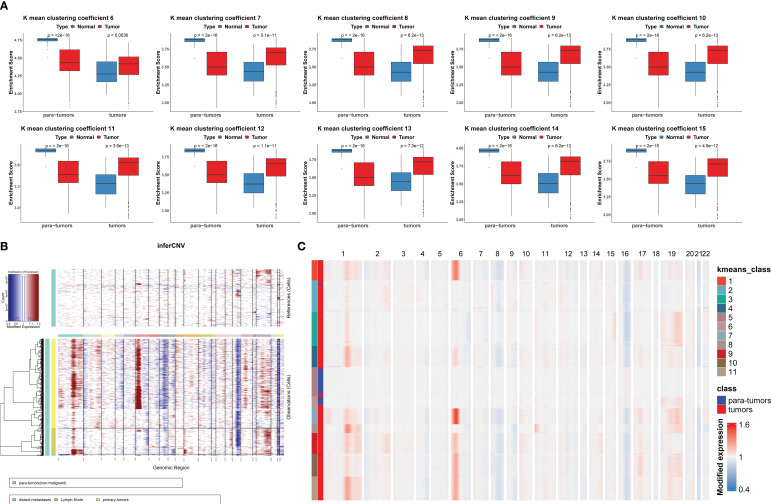
The copy number variation (CNV) profile analysis distinguishing malignant thyroid epithelial cells (mTEs) and non-malignant thyroid epithelial cells (nTEs). **(A)** The differentially expressed genes of mTEs and nTEs obtained at different *K* values were used as the ssGSEA results of the gene set in TCGA-THCA tumor and paratumor samples. **(B)** Chromosomal CNV plots of thyroid epithelial cells. The above box was the control group. The lower box shows the tumor groups. **(C)** Clustering heatmap of CNV on 22 chromosomes at *K* = 11.

Based on the fact that PTC has abundant copy number variation, we infer chromosome copy number variation (CNV) of cells based on RNA expression profile to distinguish mTEs and nTEs. First, according to the results of cell-type annotation, all thyroid epithelial cells were extracted and the CNV of each cell was inferred by using the cells in the paratumors as the reference standard (**Figure 3B**). Then, *K*-mean clustering was used to cluster CNV profiles. To determine the optimal clustering coefficient *K*, single sample gene set enrichment analysis (ssGSEA) was conducted in the TCGA-THCA dataset with the gene set composed of the top 50 genes that were differentially expressed between mTEs and nTEs in the clustering results of each *K* value (**Figure 3A**). This was the method that was created to determine the best *K*-means clustering coefficient. When *K* = 11, the difference in the enrichment fraction between tumor and paracancer samples showed the smallest *P*-value. Therefore, we used *K* = 11 to cluster CNV profiles. *K*-means clustering subgroups 5 and 6 were nTEs, and the rest of the subgroups were mTEs (**Figure 3C**). Overall, in the original mTEs, there were 8 distant metastases, 251 lymph nodes, and 701 primary tumor mTE cells classified as paratumor nTEs, and in the original nTEs, there were 346 paratumor nTE cells classified as primary tumor mTEs.

To further investigate the function of mTEs and nTEs, these two cell subsets were reclustered. mTEs were reclustered into 14 clusters (**Supplementary Figure S1A**), and the mTE3 clusters in HT patients were significantly higher than those in non-HT patients (**Supplementary Figure S1B**). Although all mTE clusters were significantly different between HT and non-HT patients, mTE3 clusters had a significant preponderance in HT patients (**Supplementary Figure S1C**). To understand the function of each mTE cluster, GO enrichment analysis was performed. The results showed that the mTE3 cluster was mainly enriched in the thyroid hormone metabolic process and thyroid hormone generation pathways (**Supplementary Table S1**). Activation of these pathways would produce more thyroid hormones, which would inhibit the secretion of TSH and thus form a TSH-inhibited environment. One of the effective treatment methods for thyroid cancer is TSH inhibition, indicating that HT patients form a TSH-inhibited environment through the high proportion of mTE3 clusters. Moreover, the occurrence and development of thyroid cancer is delayed. After that, nTEs were reclustered into 11 clusters (**Supplementary Figure S2A**). Similar to mTEs, the proportion of nTE0 and nTE2 clusters in HT patients is significantly higher than that in non-HT patients (**Supplementary Figures S2B, C**), and these two clusters are also enriched in the thyroid hormone metabolic process and thyroid hormone generation pathways (**Supplementary Figure S2D**; **Supplementary Table S2**). The above results indicate that the TSH-inhibiting environment formed by a high proportion of mTE3, nTE0, and nTE2 clusters in HT patients has an inhibitory effect on PTC.

### 3.3 Pseudotime analysis of thyroid cells

**Figure 4:**
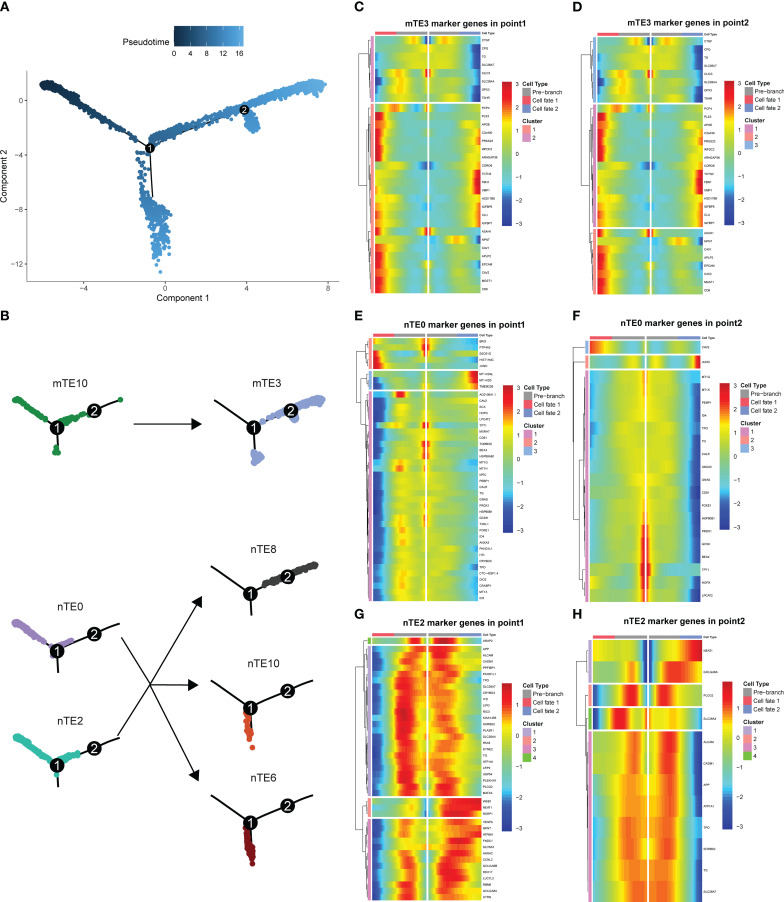
Trajectory analysis of mTE3, nTE0, and nTE2. **(A, B)** The trajectory analysis of mTE3, nTE0, and nTE2. mTE3 was from mTE10 and nTE0 and nTE2 were divided into nTE8, nTE10, and nTE6. **(C)** Heatmap showing two-gene clusters of mTE3 marker genes with different expression signatures at pseudotime point 1. **(D)** Heatmap showing three-gene clusters of mTE3 marker genes with different expression signatures at pseudotime point 2. **(E)** Heatmap showing three-gene clusters of nTE0 marker genes with different expression signatures at pseudotime point 1. **(F)** Heatmap showing three-gene clusters of nTE0 marker genes with different expression signatures at pseudotime point 2. **(G)** Heatmap showing four-gene clusters of nTE2 marker genes with different expression signatures at pseudotime point 1. **(H)** Heatmap showing four-gene clusters of nTE2 marker genes with different expression signatures at pseudotime point 2.

We next explored the mTE3, nTE0, and nTE2 cluster differentiation trajectories in HT patients by inferring the state trajectories using Monocle2. This analysis showed that nTE0 and nTE2 were at the beginning of the trajectory path, whereas mTE3 was at a terminal state (**Figures 4A, B**). Furthermore, mTE3 is mainly derived from mTE10, and nTE0 and nTE2 are mainly differentiated into nTE6, nTE8, and nTE10 (**Figure 4B**). At time transition point 1, the characteristic genes of mTE3 clusters were more likely to change from low expression to high expression, while the characteristic genes of nTE0 and nTE2 clusters were more likely to change from high expression to low expression, which was also found at time transition point 2, which further emphasized the results that nTE0 and nTE2 clusters were in the early stage of differentiation and mTE3 clusters were in the late stage of differentiation (**Figures 4C–H**). These results indicate that in HT patients, nTE0 and nTE2 clusters may differentiate into mTE3 clusters, but some cells transform into other nTE clusters, which does not affect the environment of TSH inhibition, because mTE10 clusters will differentiate into mTE3 clusters, making up for the increase of TSH caused by the decrease of nTE0 and nTE2. This dynamic transformation creates a TSH-inhibiting microenvironment that effectively inhibits PTC in HT patients.

### 3.4 Different tumor immune microenvironments between HT and non-HT patients

**Figure 5:**
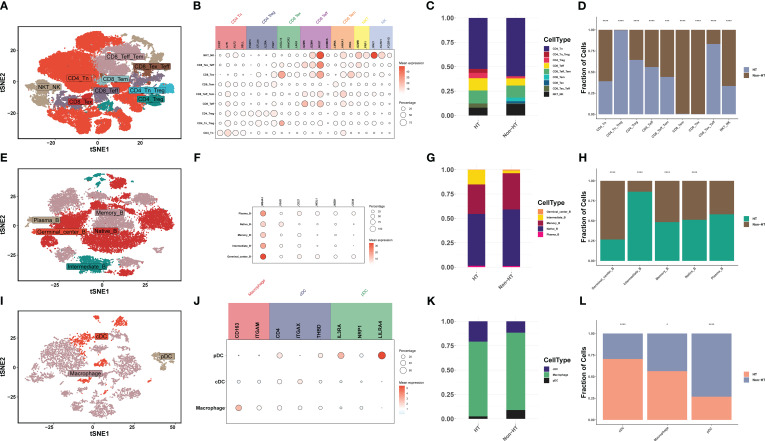
The scRNA-seq landscape of immune cells. **(A)** t-SNE plot visualizing 28 distinct clusters of T/NK cells, colored by cell type (*n* = 9). **(B)** Bubble plots showing the expression levels of marker genes for each cell type. **(C)** Horizontal bar charts showing the relative abundance of various cell types between HT and non-HT. **(D)** *Post-hoc* analysis of each cell type between HT and non-HT. **(E)** t-SNE plot visualizing 20 distinct clusters of B cells, colored by cell type (*n* = 5). **(F)** Bubble plots showing the expression levels of marker genes for each cell type. **(G)** Horizontal bar charts showing the relative abundance of various cell types between HT and non-HT. **(H)** *Post-hoc* analysis of each cell type between HT and non-HT. **(I)** t-SNE plot visualizing 20 distinct clusters of myeloid cells, colored by cell type (*n* = 3). **(J)** Bubble plots showing the expression levels of marker genes for each cell type. **(K)** Horizontal bar charts showing the relative abundance of various cell types between HT and non-HT. **(L)** *Post-hoc* analysis of each cell type between HT and non-HT. *P< 0.05, **P< 0.01, ***P< 0.001, ****P< 0.0001.

Innate immunity and adaptive immunity play important roles in the development of PTC (34). By clustering T/NK cells, B cells, and myeloid cells and counting the differences in the proportion of immune cells, differences in the tumor immune microenvironment (TIM) between HT and non-HT patients were discovered. First, T/NK cells were divided into nine cell types: 1) CD4+ T-cell subsets (*n* = 3), including naive T (Tn), regulatory T (Treg), and Tn___Treg; 2) CD8+ T-cell subsets (*n* = 5), including effector T (Teff), exhausted T (Tex), effector memory T (Tem), Tex_Teff, and Teff_Tem cells; and 3) NK cell subsets (*n* = 1), including NKT_NK cells (**Figures 5A, B**). The high proportion of CD4_Tn_Treg and CD8_Tex_Teff in HT patients indicates the activation of the immune system (**Figures 5C, D**). More CD8_Teff and less CD8_Tex can effectively mobilize the immune system to kill PTC, while the high percentage of CD4_Tn_Treg can prevent the excessive activation of the immune system and thus maintain immune homeostasis (35). Second, B cells were divided into five cell types, namely, Plasma_B (MZB1, CD38), Native_B (MS4A1, IGHD), Memory_B (MS4A1, CD27), Intermediate_B (IGHD, CD27) and Germinal_center_B (MS4A1, NEIL1) (**Figures 5E, F**). Intermediate_B was the only cell with a significantly high proportion in HT patients (**Figures 5G, H**). Myeloid cells were divided into three cell types, and macrophage cells are the primary type (**Figures 5I, J**). However, high infiltration of conventional dendritic cells (cDCs) in HT patients predicted stronger activation of T cells. Although plasmacytoid dendritic cells (pDCs) could also activate T cells, their activation capacity was smaller (**Figures 5K, L**). The above proportion of immune cells indicates that HT patients can better activate their own immune system, and the presence of PTC prevents excessive activation of the immune system to reach homeostasis, which forms a dynamic balance between autoimmune diseases and cancer. This finding enables us to clearly see that HT inhibits the development of PTC by mobilizing CD4_Tn_Treg, CD8_Tex_Teff, Intermediate_B, and cDC.

### 3.5 Identification of diverse subtypes of stromal cells

**Figure 6:**
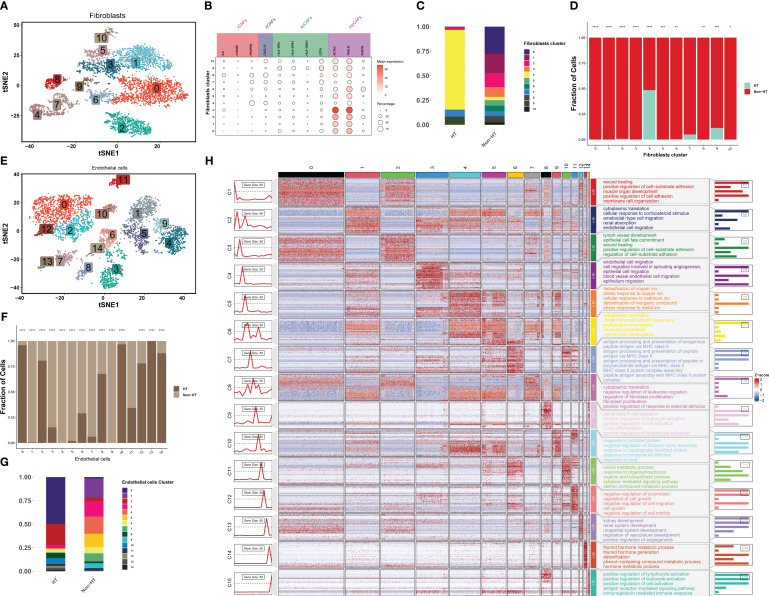
The scRNA-seq landscape of stromal cells. **(A)** t-SNE plot visualizing 11 distinct clusters of fibroblasts, colored by cell type (*n* = 4). **(B)** Bubble plots showing the expression levels of marker genes for each cell type. **(C)** Horizontal bar charts showing the relative abundance of various cell types between HT and non-HT. **(D)** *Post-hoc* analysis of each cell type between HT and non-HT. **(E)** t-SNE plot showing endothelial cells colored by clusters (*n* = 15). **(F)** Horizontal bar charts showing the relative abundance of various clusters between HT and non-HT. **(G)** *Post-hoc* analysis of each cluster between HT and non-HT. **(H)** GO enrichment analysis of the top 50 marker genes in 15 clusters. *P< 0.05, **P< 0.01, ***P< 0.001, ****P< 0.0001.

Stromal cells are mainly composed of fibroblasts and endothelial cells. The fibroblasts were divided into 11 cell clusters (**Figure 6A**), which were cell-type-annotated according to the characteristic genes of the four cancer-associated fibroblasts (CAFs) (36). The characteristic genes of myCAFs were highly expressed in most cell subsets, but iCAFs and irCAFs were highly expressed in cell clusters 4, 7, and 8 (**Figure 6B**). In particular, cell cluster 4 is an important component of CAF in HT patients, and the expression level of PDGFRA, a marker gene of iCAFs, is the highest (**Figures 6B–D**). The inflammatory and immune environments formed by iCAFs and irCAFs indicate that CAF in HT patients is more benign, which is superior to myCAF’s role in tissue repair during cancer development. Furthermore, endothelial cells were divided into 15 cell clusters (**Figure 6E**), and endothelial cell cluster 13 almost only existed in HT patients (**Figures 6F, G**). In order to understand its biological function, GO enrichment analysis was conducted on all endothelial cell clusters, and consistent with nTE0, nTE2, and mTE3 clusters, the marker gene of endothelial cell cluster 13 was mainly enriched in the thyroid hormone metabolic process and thyroid hormone generation pathways (**Figure 6H**; **Supplementary Table S3**). In conclusion, a high proportion of fibroblast cluster 4 and endothelial cell cluster 13 in stromal cells is a major feature of HT patients, which will better inhibit the development of PTC.

### 3.6 Multiple cell crosstalk reveals the regulatory mechanism of tumor microenvironment

**Figure 7:**
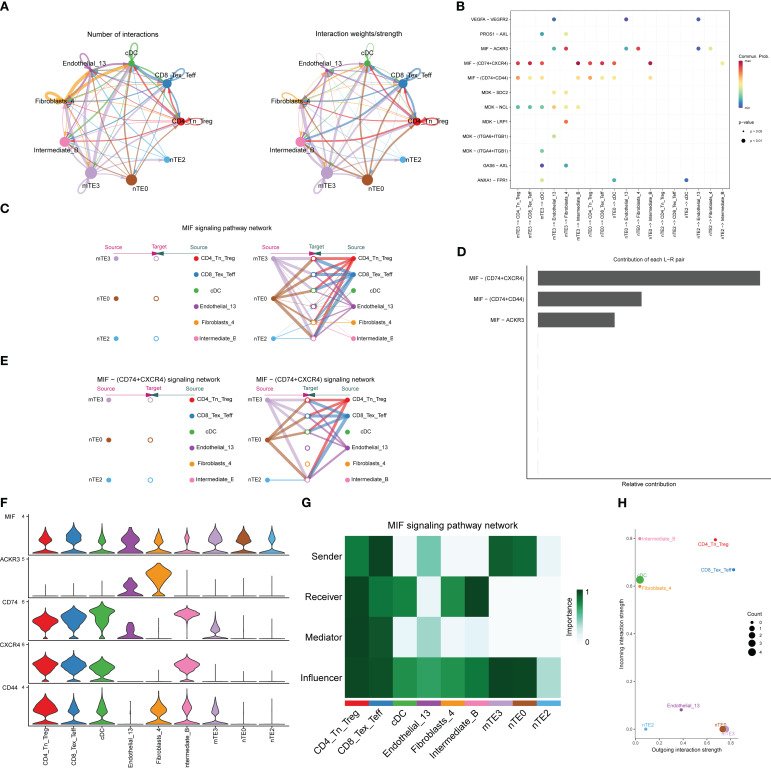
Cell–cell communication analysis of HASCs. **(A)** Net count map of HASC interactions. HASC interaction weight map. The thicker the line, the greater the number of interactions and the greater the weight/intensity of interactions between the two cell types. **(B)** The significantly related ligand–receptor interactions from the main thyroid epithelial cells to other immune and stromal cells. **(C)** Hierarchy plot of interactions between selected target cells and other cells in the MIF signaling pathway network. **(D)** Relative contribution of each ligand–receptor pair to the overall communication network of signaling pathways, which is the ratio of the total communication probability of the inferred network of each ligand–receptor pair to that of the signaling pathways. **(E)** Hierarchy plot of interactions between selected target cells and other cells in the MIF–(CD74+CXCR4) signaling network. **(F)** Violin plot showing the expression patterns of signaling genes involved in the inferred signaling network. **(G)** The network centrality index of each cell population was calculated to identify the role of each type of cell in the MIF signaling pathway. **(H)** Scatter plot visualizing the dominant sender (source) and receiver (target) in the MIF signaling pathway in 2D space.

Through the above systematic analysis, we found that nTE0, nTE2, and mTE3 contents were abundant in HT patients, and the environment that causes TSH inhibition can effectively control the development of PTC. At the same time, it was noticed that the tumor microenvironment of HT patients has a significantly high proportion of CD4_Tn_Treg, CD8_Tex_Teff, Intermediate_B, cDC, fibroblast cluster 4, and endothelial cell cluster 13, indicating that there may be a cross-talk among these cells. We refer to these cells as HASCs. To further understand the underlying regulatory mechanisms, we used CellChat to infer intercellular communication between nTE0, nTE2, and mTE3 and other cell types based on the ligand–receptor (L–R). mTE3 and nTE0 interact more closely with other cells, both in terms of the number and intensity of interactions, and more as senders of cell communication. On the other hand, immune and stromal cells interact with other cells more as receivers of cell communication (**Figure 7A**). nTE0, nTE2, and mTE3 interact primarily with MIF signaling pathways mediated by CD74 and CXCR4 receptors on immune and stromal cells via MIF ligands (**Figure 7B**). In the MIF signaling pathway network, nTE0, nTE2, and mTE3 showed a similar interaction relationship with all other cells, that is, nTE0 and mTE3 had interactions with all other cells (**Figure 7C**), and MIF–(CD74+CXCR4) was dominant in these interactions (**Figure 7D**). Further studies showed that in the MIF–(CD74+CXCR4) signaling pathway, CD4_Tn_Treg, CD8_Tex_Teff, cDC, and Intermediate_B interact with many other cells in the signaling network (**Figure 7E**), which can also be seen by the expression value of the L–R pairs (**Figure 7F**). To further determine the role of these cells in the MIF signaling pathway, a cellular communication network system analysis was performed. The results were consistent with the previous results: nTE0 and mTE3 were mainly signal transmitters, while all the other cells except E were receivers, and CD4_Tn_Treg and CD8_Tex_Teff were very active, playing the four roles of signal sending, receiving, mediating, and influencing (**Figure 7G**). To more intuitively define the role of all cells in the MIF signaling pathway, we visualized the dominant sender (source) and receiver (target) in 2D space (**Figure 7H**). There was no doubt that nTE0 and mTE3 were the senders of the signal; Intermediate_B, cDC, and fibroblast cluster 4 were the receivers of the signal; CD4_Tn_Treg, endothelial cell cluster 13, and CD8_Tex_Teff were both the sender and the receiver; and nTE2 was almost neither the sender nor the receiver.

### 3.7 Relationship between HASC infiltration and clinical features in bulk RNA-seq

**Figure 8:**
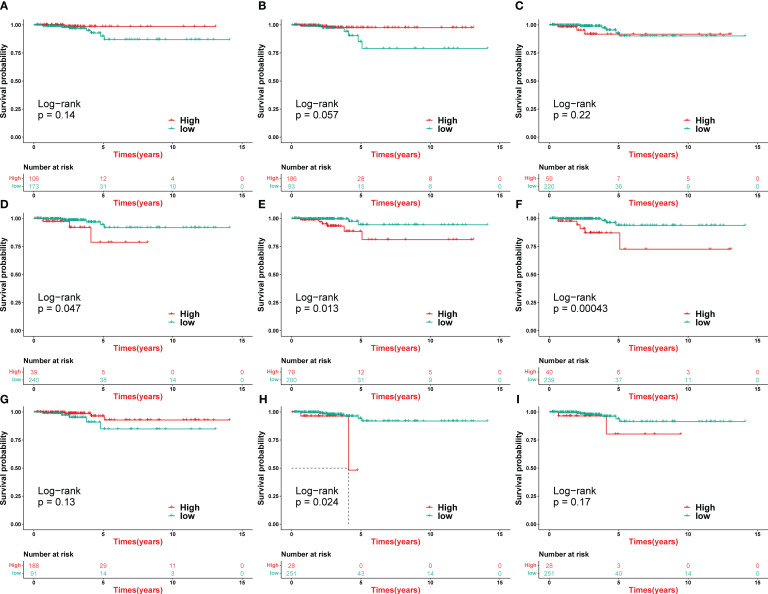
Survival analysis of HASCs in TCGA-THCA. **(A–I)** Kaplan–Meier curve of OS according to CD4_Tn_Treg (log-rank test: *P* = 0.14) **(A)**, CD8_Tex_Teff (log-rank test: *P* = 0.057) **(B)**, cDC (log-rank test: *P* = 0.22) **(C)**, endothelial cell cluster 13 (log-rank test: *P* = 0.047) **(D)**, fibroblast cluster 4 (log-rank test: *P* = 0.013) **(E)**, Intermediate_B (log-rank test: *P* = 0.00043) **(F)**, mTE3 (log-rank test: *P* = 0.13) **(G)**, nTE0 (log-rank test: *P* = 0.024) **(H)**, and nTE2 (log-rank test: *P* = 0.17) **(I)**.

First, we investigated the association of HASC infiltration with PTC prognosis. Patients in groups CD4_Tn_Treg, CD8_Tex_Teff, and mTE3 with high infiltration had a better prognosis although survival differences were not significant (*P* > 0.05) (**Figures 8A, B, G**), which was consistent with previous intercellular communication results that CD4_Tn_Treg and CD8_Tex_Teff play multiple roles in critical cell communication. Since the environment of TSH suppression was mainly created by the high infiltration of mTE3, nTE0, and nTE2, it was logical that patients in the high infiltration group of mTE3 would have a better prognosis, but we observed a paradoxical phenomenon that patients with high infiltration of nTE0 and nTE2 would have a worse prognosis (**Figures 8H, I**). Through the previous trajectory analysis, we found that nTE0 and nTE2 were in the early stage of differentiation, while mTE3 was in the late stage of differentiation. The high infiltration of nTE0 and nTE2 implies that cell differentiation had not begun or was just beginning when the characteristic genes of nTE0 and nTE2 were not immediately functional and the TSH-suppressive environment had not yet formed. In contrast, the high infiltration of mTE3 cells indicated that cell differentiation was nearing completion, the signature genes of mTE3 had completed their role in thyroid hormone production, and the TSH-suppressed environment effectively prolonged patient survival. The high infiltration of cDC, fibroblast cluster 4, and Intermediate_B as receivers of cell communication meant that cell differentiation had fully started, the TSH-suppressive environment had been established, and the patient’s prognosis was naturally better (**Figures 8C, E, F**). As a group of tissues that can phagocytose foreign bodies, bacteria, necrosis, and aging and participate in the immune activities of the body, patients with high infiltration of endothelial cell cluster 13 would have a better survival time (**Figure 8D**). Then, we examined the association of HASCs with clinical features. mTE3 was significantly enriched in patients younger than 60, validating the results of the survival analysis (**Supplementary Figure S3A**). Significant differences in terms of gender were found in CD4_Tn_Treg and Intermediate_B, where CD4_Tn_Treg was significantly enriched in female patients, while Intermediate_B was significantly enriched in male patients (**Supplementary Figure S3B**). In terms of tumor metastasis, CD8_Tex_Teff and cDC were enriched in patients without metastasis, revealing their role in preventing tumor metastasis (**Supplementary Figure S3C**). Most of the HASCs were significantly differentially enriched in the presence or absence of regional lymph node metastasis, indicating that regional lymph node metastasis is an important feature of PTC (**Supplementary Figure S3D**). The results of tumor T stage and AJCC stage showed the same enrichment trend of HASCs. The changes of mTE3, nTE0, and nTE2 were not obvious, but the immune cells CD4_Tn_Treg, CD8_Tex_Teff, and cDC showed a fluctuating change trend, that was from high to low (**Supplementary Figures S3E, F**), which reflected the dynamic changes of immune cells in the development of cancer. However, advanced patients usually have fewer immune cells, which is consistent with some existing studies (37).

### 3.8 Consensus clustering of TCGA-THCA based on HASCs

**Figure 9:**
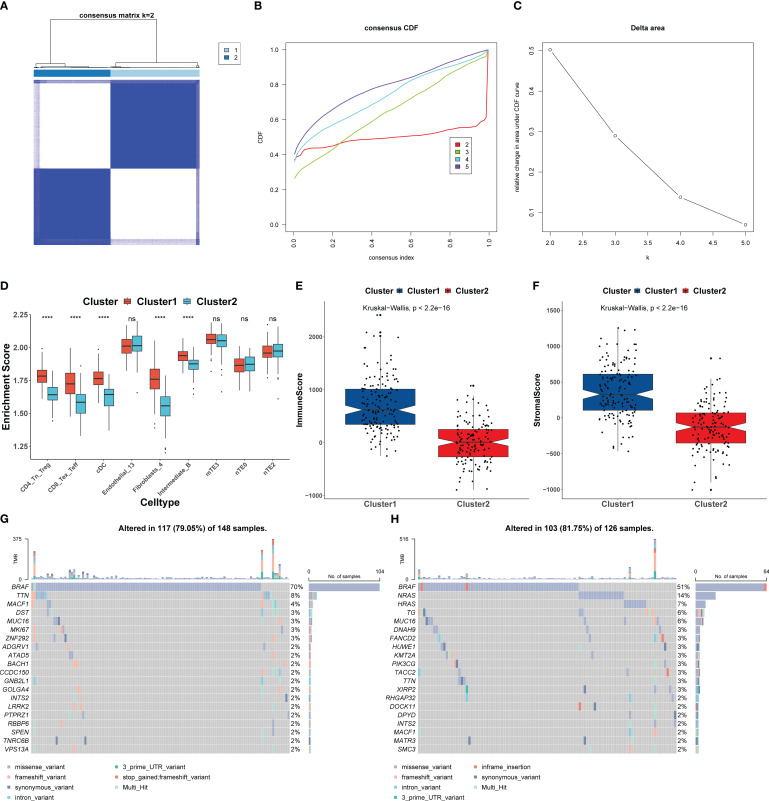
Identification of different molecular subtypes based on HASCs. **(A)** Consensus clustering matrix when *K* = 2. **(B)** Consensus clustering CDF with *K* values of 2 to 9. **(C)** Relative change in area under the CDF curve for *K* = 2. **(D)** Box plot of the HASC content between cluster 1 and cluster 2. **(E, F)** Box plot of the difference between immune scores **(E)** and stromal scores **(F)** in subtypes based on the ESTIMATE algorithm. **(G, H)** SNV waterfall of the top 20 (mutation frequency) genes in cluster 1 (*n* = 148) **(G)** and cluster 2 (*n* = 126) **(H)**. *P< 0.05, **P< 0.01, ***P< 0.001, ****P< 0.0001.

**Figure 10:**
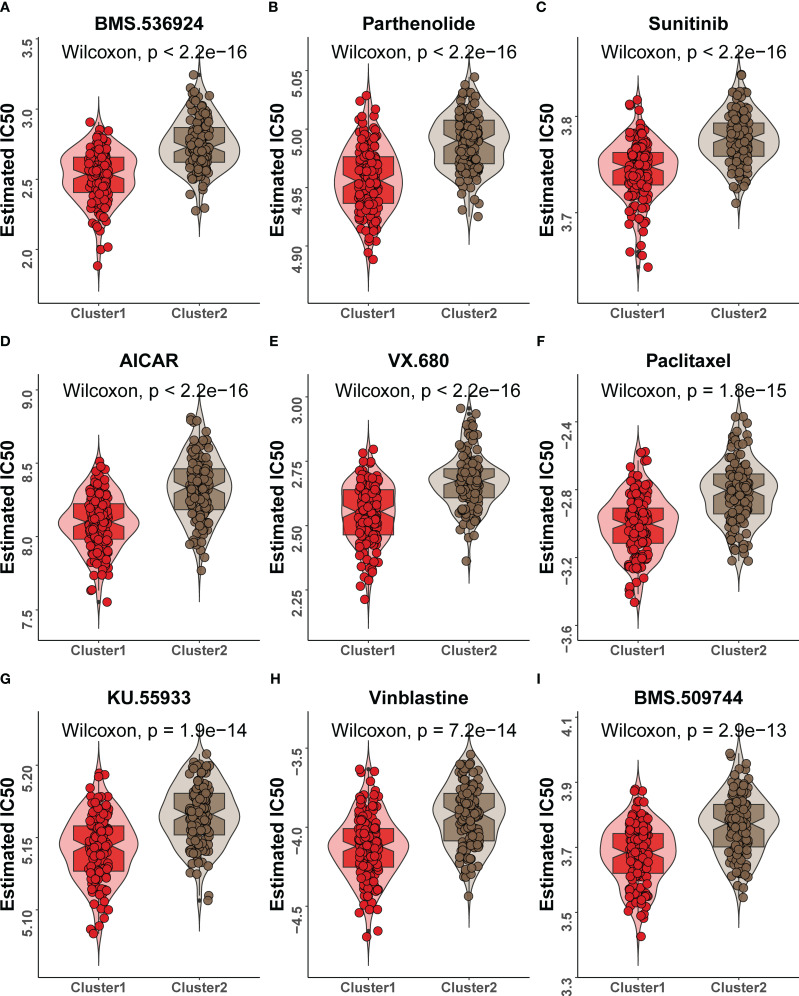
Variations in drug sensitivity between cluster 1 and cluster 2. **(A–I)** IC50 box diagram of the nine drugs with significant difference in drug sensitivity in cluster 1 and cluster 2, respectively, in which red indicated cluster 1 and brown indicated cluster 2.

The HASCs were further utilized for consensus clustering analysis. When the clustering coefficient *K* = 2, the clustering effect was the best, and the internal consistency and stability of the subgroups were good (**Figures 9A–C**). Cluster 1 was more abundant in immune cells, in contrast to cluster 2, which was more abundant in stromal cells (**Figure 9D**). This finding was validated by the results of sample immune scoring and stromal scoring evaluated by the ESTIMATE algorithm (**Figures 9E, F**). Previous studies have confirmed that high infiltration of immune cells such as CD8+ T cells and CD4+ T cells predicts better prognosis. Next, we compared the mutation status of cluster 1 and cluster 2 and found that the overall tumor mutation burden (TMB) of cluster 1 was higher, indicating that cluster 1 could benefit better from immunotherapy (38). At the same time, we found that the BRAF gene mutation frequency was the highest in both subgroups, which was consistent with previous studies (6). However, we also found that cluster 1 and cluster 2 showed different mutation patterns. Mutations in cluster 1 mainly occurred in TTN and MACF1 genes, while mutations in cluster 2 mainly occurred in NRAS and HRAS genes of the RAS gene family (**Figures 9G, H**). The IC50 value of 138 drugs in the Genomics of Drug Sensitivity in Cancer (GDSC) database was predicted based on the expression profile of TCGA-THCA. The top 9 drugs with significant differences in drug sensitivity between subgroups were shown here, which were BMS.536924 (**Figure 10A**), parthenolide (**Figure 10B**), sunitinib (**Figure 10C**), AICAR (**Figure 10D**), VX.680 (**Figure 10E**), paclitaxel (**Figure 10F**), KU.55933 (**Figure 10G**), vinblastine (**Figure 10H**), and BMS.509744 (**Figure 10I**). Among them, sunitinib, VX.680, paclitaxel, and vinblastine are anticancer drugs, and cluster 1 showed a stronger drug sensitivity to these drugs, indicating that this cluster had a better response to drug treatment, which was consistent with the previous results of higher TMB. The classification of molecular subtypes in TCGA-THCA samples allows us to more precisely target drug therapy, and this new finding will help in the treatment of PTC patients.

## 4 Discussion

The present investigation addresses a critical gap in our understanding of PTC by leveraging scRNA-seq, transcending the limitations of conventional bulk RNA-seq methodologies that inadequately delineate cellular heterogeneity (39, 40). By harnessing the high-resolution capabilities of scRNA-seq, we align with prior research highlighting its significance in PTC exploration (41), offering a refined perspective on the disease. Given the autoimmune nature of HT and its potential to modulate immune cell activity—a pivotal factor in PTC management (42)—our study underscores the necessity to elucidate HT’s impact on PTC.

Our work innovates in the approach to distinguish malignant from non-malignant cells, a challenge traditionally addressed through inferential CNV analyses in cancer research (25, 43–48). To overcome the impediment of low CNV variability in PTC, we employed *K*-means clustering informed by the statistical significance of differential enrichment scores derived from TCGA data. This novel methodology optimizes CNV-based classification, contributing a robust tool for future scRNA-seq studies.

A pivotal discovery lies in the identification of HT-associated specific cell populations (HASCs). Our findings resonate with the therapeutic efficacy of TSH suppression in PTC management (49), revealing that HASC subsets—marked by mTE3, nTE0, and nTE2 cells enriched in thyroid hormone pathways—are conducive to a TSH-suppressive milieu, thereby affirming HT’s positive influence on PTC progression through these cell clusters (50).

Additionally, our study elucidates the intricate interplay between immune and stromal cells with thyroid cells, pinpointing specific cell clusters such as CD4+ Tn Tregs, CD8+ Teff, and others, where the MIF–(CD74+CXCR4) axis emerges as a crucial mediator. This pathway, previously implicated in PTC immunotherapy (51), highlights immune cells’ centrality in TSH milieu regulation, underscoring their potential as therapeutic targets. Notably, CD4_Tn_Treg and CD8_Tex_Teff cell subsets were found to play multiple roles in the cellular communication of HASCs, which was consistent with previous studies on the role of T cells in PTC (24).

A preceding meta-analysis has affirmed the differential impacts of immune and stromal cells in the tumor microenvironment (50). Consequently, we ventured to elucidate the prognostic implications of HASCs at the tissue level. Our findings revealed heterogeneous effects of individual cell types on disease prognosis, with CD4+ Tn Tregs, CD8+ Tex Teffs, and mTE3 exhibiting elevated enrichment in M0, T1, and stage I, concurrently associated with younger patient age. No discernible variation was noted concerning gender or N-stage classification. This cellular heterogeneity underscores the complexity of tumor ecosystems, a characteristic well-documented in TCGA-THCA cohorts (51). To further dissect this heterogeneity, molecular stratification emerges as the premier strategy, endorsed extensively in the literature. Thus, consensus clustering was employed to segregate TCGA-THCA cases into two distinct clusters (cluster 1 and cluster 2), where cluster 1 displayed heightened HASC enrichment, indicative of a correlation with HT, an observation corroborated by the ESTIMATE algorithm. Additionally, our investigation of drug responsiveness revealed cluster 1 to be more susceptible to chemotherapeutic agents like sunitinib, VX-680, paclitaxel, and vinblastine, reinforcing the hypothesis that cluster 1 represents HT-positive PTC, with its enriched immune landscape enhancing sensitivity to anticancer therapies, a pivotal insight for therapeutic strategies.

In aggregate, our research constitutes a comprehensive exploration of cellular subset disparities between HT-positive and HT-negative PTC patients at the single-cell resolution. By isolating HASCs from differential cell populations, we facilitated an in-depth examination of intercellular communication dynamics, unearthing regulatory mechanisms. Expanding upon prior studies, we quantified HASC abundance in bulk transcriptomic datasets and conducted cluster analysis on TCGA samples. This work underscores the significance of HT in modulating PTC progression and identifies the MIF–(CD74+CXCR4) axis as a potential therapeutic target. While acknowledging limitations, our study undeniably illuminates the favorable influence of HT on PTC outcomes, thereby furnishing a fresh perspective and theoretical foundation for subsequent inquiries.

## Data availability statement

The original contributions presented in the study are included in the article/**Supplementary Material**. Further inquiries can be directed to the corresponding authors.

## Author contributions

HM: Conceptualization, Data curation, Writing – review & editing. GL: Investigation, Methodology, Resources, Validation, Visualization, Writing – original draft, Writing – review & editing. DH: Conceptualization, Writing – original draft. YGS: Resources, Writing – original draft. QJ: Conceptualization, Writing – original draft. YL: Conceptualization, Writing – original draft. YS: Conceptualization, Writing – original draft. DZ: Conceptualization, Writing – original draft. XC: Conceptualization, Writing – original draft.

## References

[1] Pellegriti G Frasca F Regalbuto C Squatrito S Vigneri R. Worldwide increasing incidence of thyroid cancer: update on epidemiology and risk factors. J Cancer Epidemiol. (2013) 2013:965212. doi: 10.1155/2013/965212 23737785 PMC3664492

[2] Bray F Ferlay J Soerjomataram I Siegel RL Torre LA Jemal A. Global cancer statistics 2018: GLOBOCAN estimates of incidence and mortality worldwide for 36 cancers in 185 countries. CA Cancer J Clin. (2018) 68:394–424. doi: 10.3322/caac.21492 30207593

[3] Kwak JY Kim EK Kim JK Han JH Hong SW Park TS. Dual priming oligonucleotide-based multiplex PCR analysis for detection of BRAFV600E mutation in FNAB samples of thyroid nodules in BRAFV600E mutation-prevalent area. Head Neck. (2010) 32:490–8. doi: 10.1002/hed.21210 19672964

[4] Tiedje V Fagin JA. Therapeutic breakthroughs for metastatic thyroid cancer. Nat Rev Endocrinol. (2020) 16:77–8. doi: 10.1038/s41574-019-0307-2 PMC7470005 31819229

[5] Mehnert JM Varga A Brose MS Aggarwal RR Lin CC Prawira A. Safety and antitumor activity of the anti-PD-1 antibody pembrolizumab in patients with advanced, PD-L1-positive papillary or follicular thyroid cancer. BMC Cancer. (2019) 19:196. doi: 10.1186/s12885-019-5380-3 30832606 PMC6399859

[6] Kebebew E Weng J Bauer J Ranvier G Clark OH Duh QY. The prevalence and prognostic value of BRAF mutation in thyroid cancer. Ann Surg. (2007) 246:466–70. doi: 10.1097/SLA.0b013e318148563d PMC1959359 17717450

[7] Gooden MJ Bock GH Leffers N Daemen T Nijman HW. The prognostic influence of tumour-infiltrating lymphocytes in cancer: a systematic review with meta-analysis. Br J Cancer. (2011) 105:93–103. doi: 10.1038/bjc.2011.189 21629244 PMC3137407

[8] Gentles AJ Newman AM Liu CL Bratman SV Feng W Kim D. The prognostic landscape of genes and infiltrating immune cells across human cancers. Nat Med. (2015) 21:938–45. doi: 10.1038/nm.3909 PMC4852857 26193342

[9] Gabrilovich DI Nagaraj S. Myeloid-derived suppressor cells as regulators of the immune system. Nat Rev Immunol. (2009) 9:162–74. doi: 10.1038/nri2506 PMC2828349 19197294

[10] Jung KY Cho SW Kim YA Kim D Oh BC Park DJ. Cancers with higher density of tumor-associated macrophages were associated with poor survival rates. J Pathol Transl Med. (2015) 49:318–24. doi: 10.4132/jptm.2015.06.01 PMC4508569 26081823

[11] Lee EK Sunwoo JB. Natural killer cells and thyroid diseases. Endocrinol Metab (Seoul). (2019) 34:132–7. doi: 10.3803/EnM.2019.34.2.132 PMC6599908 31257741

[12] Gupta S Patel A Folstad A Fenton C Dinauer CA Tuttle RM. Infiltration of differentiated thyroid carcinoma by proliferating lymphocytes is associated with improved disease-free survival for children and young adults. J Clin Endocrinol Metab. (2001) 86:1346–54. doi: 10.1210/jc.86.3.1346 11238531

[13] Ahn D Heo SJ Park JH Kim JH Sohn JH Park JY. Clinical relationship between Hashimoto's thyroiditis and papillary thyroid cancer. Acta Oncol. (2011) 50:1228–34. doi: 10.3109/0284186X.2011.602109 21871002

[14] Konturek A Barczyński M Wierzchowski W Stopa M Nowak W. Coexistence of papillary thyroid cancer with Hashimoto thyroiditis. Langenbecks Arch Surg. (2013) 398:389–94. doi: 10.1007/s00423-012-1021-x PMC3597286 23099542

[15] Lee JH Kim Y Choi JW Kim YS. The association between papillary thyroid carcinoma and histologically proven Hashimoto's thyroiditis: a meta-analysis. Eur J Endocrinol. (2013) 168:343–9. doi: 10.1530/EJE-12-0903 23211578

[16] Zhang Y Dai J Wu T Yang N Yin Z. The study of the coexistence of Hashimoto's thyroiditis with papillary thyroid carcinoma. J Cancer Res Clin Oncol. (2014) 140:1021–6. doi: 10.1007/s00432-014-1629-z PMC11823965 24619663

[17] Resende de Paiva C Grønhøj C Feldt-Rasmussen U von Buchwald C. Association between Hashimoto's thyroiditis and thyroid cancer in 64,628 patients. Front Oncol. (2017) 7:53. doi: 10.3389/fonc.2017.00053 28443243 PMC5385456

[18] Peng M Wei G Zhang Y Li H Lai Y Guo Y. Single-cell transcriptomic landscape reveals the differences in cell differentiation and immune microenvironment of papillary thyroid carcinoma between genders. Cell Biosci. (2021) 11:39. doi: 10.1186/s13578-021-00549-w 33588924 PMC7885238

[19] Ceolin L Siqueira DR Romitti M Ferreira CV Maia AL. Molecular basis of medullary thyroid carcinoma: the role of RET polymorphisms. Int J Mol Sci. (2012) 13:221–39. doi: 10.3390/ijms13010221 PMC3269683 22312249

[20] Mohammadi M Hedayati M. A brief review on the molecular basis of medullary thyroid carcinoma. Cell J. (2017) 18:485–92. doi: 10.22074/cellj.2016.4715 PMC5086327 28042533

[21] Luo H Xia X Kim GD Liu Y Xue Z Zhang L. Characterizing dedifferentiation of thyroid cancer by integrated analysis. Sci Adv. (2021) 7. doi: 10.1126/sciadv.abf3657 PMC8318367 34321197

[22] Barrett T Wilhite SE Ledoux P Evangelista C Kim IF Tomashevsky M. NCBI GEO: archive for functional genomics data sets–update. Nucleic Acids Res. (2013) 41:D991–5. doi: 10.1093/nar/gks1193 PMC3531084 23193258

[23] Stuart T Butler A Hoffman P Hafemeister C Papalexi E Mauck WM. Comprehensive integration of single-cell data. Cell. (2019) 177:1888–902. doi: 10.1016/j.cell.2019.05.031 PMC6687398 31178118

[24] Pu W Shi X Yu P Zhang M Liu Z Tan L. Single-cell transcriptomic analysis of the tumor ecosystems underlying initiation and progression of papillary thyroid carcinoma. Nat Commun. (2021) 12:6058. doi: 10.1038/s41467-021-26343-3 34663816 PMC8523550

[25] Patel AP Tirosh I Trombetta JJ Shalek AK Gillespie SM Wakimoto H. Single-cell RNA-seq highlights intratumoral heterogeneity in primary glioblastoma. Science. (2014) 344:1396–401. doi: 10.1126/science.1254257 PMC4123637 24925914

[26] Qiu X Hill A Packer J Lin D Ma YA Trapnell C. Single-cell mRNA quantification and differential analysis with Census. Nat Methods. (2017) 14:309–15. doi: 10.1038/nmeth.4150 PMC5330805 28114287

[27] Jin S Guerrero-Juarez CF Zhang L Chang I Ramos R Kuan CH. Inference and analysis of cell-cell communication using CellChat. Nat Commun. (2021) 12:1088. doi: 10.1038/s41467-021-21246-9 33597522 PMC7889871

[28] Goldman MJ Craft B Hastie M Repecka K McDade F Kamath A. Visualizing and interpreting cancer genomics data via the Xena platform. Nat Biotechnol. (2020) 38:675–8. doi: 10.1038/s41587-020-0546-8 PMC7386072 32444850

[29] Wilkerson MD Hayes DN. ConsensusClusterPlus: a class discovery tool with confidence assessments and item tracking. Bioinformatics. (2010) 26:1572–3. doi: 10.1093/bioinformatics/btq170 PMC2881355 20427518

[30] Aran D Sirota M Butte AJ. Systematic pan-cancer analysis of tumour purity. Nat Commun. (2015) 6:8971. doi: 10.1038/ncomms9971 26634437 PMC4671203

[31] Zeng D Ye Z Shen R Yu G Wu J Xiong Y. IOBR: multi-omics immuno-oncology biological research to decode tumor microenvironment and signatures. Front Immunol. (2021) 12:687975. doi: 10.3389/fimmu.2021.687975 34276676 PMC8283787

[32] Mayakonda A Lin DC Assenov Y Plass C Koeffler HP. Maftools: efficient and comprehensive analysis of somatic variants in cancer. Genome Res. (2018) 28:1747–56. doi: 10.1101/gr.239244.118 PMC6211645 30341162

[33] Geeleher P Cox N Huang RS. pRRophetic: an R package for prediction of clinical chemotherapeutic response from tumor gene expression levels. PloS One. (2014) 9:e107468. doi: 10.1371/journal.pone.0107468 25229481 PMC4167990

[34] Ferrari SM Fallahi P Elia G Ragusa F Ruffilli I Paparo SR. Thyroid autoimmune disorders and cancer. Semin Cancer Biol. (2020) 64:135–46. doi: 10.1016/j.semcancer.2019.05.019 31158464

[35] de Candia P Procaccini C Russo C Lepore MT Matarese G. Regulatory T cells as metabolic sensors. Immunity. (2022) 55:1981–92. doi: 10.1016/j.immuni.2022.10.006 36351373

[36] Lavie D Ben-Shmuel A Erez N Scherz-Shouval R. Cancer-associated fibroblasts in the single-cell era. Nat Cancer. (2022) 3:793–807. doi: 10.1038/s43018-022-00411-z 35883004 PMC7613625

[37] Sajid A Rahman H Ambudkar SV. Advances in the structure, mechanism and targeting of chemoresistance-linked ABC transporters. Nat Rev Cancer. (2023) 23:762–779. doi: 10.1038/s41568-023-00612-3 37714963

[38] Anagnostou V Bardelli A Chan TA Turajlic S. The status of tumor mutational burden and immunotherapy. Nat Cancer. (2022) 3:652–6. doi: 10.1038/s43018-022-00382-1 35764740

[39] He J Zhou M Yin J Wan J Chu J Jia J. METTL3 restrains papillary thyroid cancer progression via m(6)A/c-Rel/IL-8-mediated neutrophil infiltration. Mol Ther. (2021) 29:1821–37. doi: 10.1016/j.ymthe.2021.01.019 PMC8116572 33484966

[40] Buj R Mallona I Díez-Villanueva A Zafon C Mate JL Roca M. Kallikreins stepwise scoring reveals three subtypes of papillary thyroid cancer with prognostic implications. Thyroid. (2018) 28:601–12. doi: 10.1089/thy.2017.0501 29635968

[41] Wang T Shi J Li L Zhou X Zhang H Zhang X. Single-cell transcriptome analysis reveals inter-tumor heterogeneity in bilateral papillary thyroid carcinoma. Front Immunol. (2022) 13:840811. doi: 10.3389/fimmu.2022.840811 35515000 PMC9065345

[42] Ehlers M Schott M. Hashimoto's thyroiditis and papillary thyroid cancer: are they immunologically linked? Trends Endocrinol Metab. (2014) 25:656–64. doi: 10.1016/j.tem.2014.09.001 25306886

[43] Chen K Wang Y Hou Y Wang Q Long D Liu X. Single cell RNA-seq reveals the CCL5/SDC1 receptor-ligand interaction between T cells and tumor cells in pancreatic cancer. Cancer Lett. (2022) 545:215834. doi: 10.1016/j.canlet.2022.215834 35917973

[44] Liu Y He S Wang XL Peng W Chen QY Chi DM. Tumour heterogeneity and intercellular networks of nasopharyngeal carcinoma at single cell resolution. Nat Commun. (2021) 12:741. doi: 10.1038/s41467-021-21043-4 33531485 PMC7854640

[45] Puram SV Tirosh I Parikh AS Patel AP Yizhak K Gillespie S. Single-cell transcriptomic analysis of primary and metastatic tumor ecosystems in head and neck cancer. Cell. (2017) 171:1611–1624 e24. doi: 10.1016/j.cell.2017.10.044 29198524 PMC5878932

[46] Müller S Liu SJ Di Lullo E Malatesta M Pollen AA Nowakowski TJ. Single-cell sequencing maps gene expression to mutational phylogenies in PDGF- and EGF-driven gliomas. Mol Syst Biol. (2016) 12:889. doi: 10.15252/msb.20166969 27888226 PMC5147052

[47] Tirosh I Venteicher AS Hebert C Escalante LE Patel AP Yizhak K. Single-cell RNA-seq supports a developmental hierarchy in human oligodendroglioma. Nature. (2016) 539:309–13. doi: 10.1038/nature20123 PMC5465819 27806376

[48] Chen YP Yin JH Li WF Li HJ Chen DP Zhang CJ. Single-cell transcriptomics reveals regulators underlying immune cell diversity and immune subtypes associated with prognosis in nasopharyngeal carcinoma. Cell Res. (2020) 30:1024–42. doi: 10.1038/s41422-020-0374-x PMC7784929 32686767

[49] Gild ML Bullock M Robinson BG Clifton-Bligh R. Multikinase inhibitors: a new option for the treatment of thyroid cancer. Nat Rev Endocrinol. (2011) 7:617–24. doi: 10.1038/nrendo.2011.141 21862995

[50] Xu J Ding K Mu L Huang J Ye F Peng Y. Hashimoto's thyroiditis: A "Double-edged sword" in thyroid carcinoma. Front Endocrinol (Lausanne). (2022) 13:801925. doi: 10.3389/fendo.2022.801925 35282434 PMC8907134

[51] Minna E Romeo P Dugo M De Cecco L Todoerti K Pilotti S. Correction: miR-451a is underexpressed and targets AKT/mTOR pathway in papillary thyroid carcinoma. Oncotarget. (2018) 9:12534. doi: 10.18632/oncotarget.v9i15 29552331 PMC5844767

