# Supplementary material for: Impact of Hashimoto’s thyroiditis on the tumor microenvironment in papillary thyroid cancer: insights from single-cell analysis
Source: Front Endocrinol (Lausanne). 2024 Sep 16;15:1339473. doi: 10.3389/fendo.2024.1339473 (PMC11439672; doi:10.3389/fendo.2024.1339473)
Supplement: Supplementary file 1 [file DataSheet1.docx]

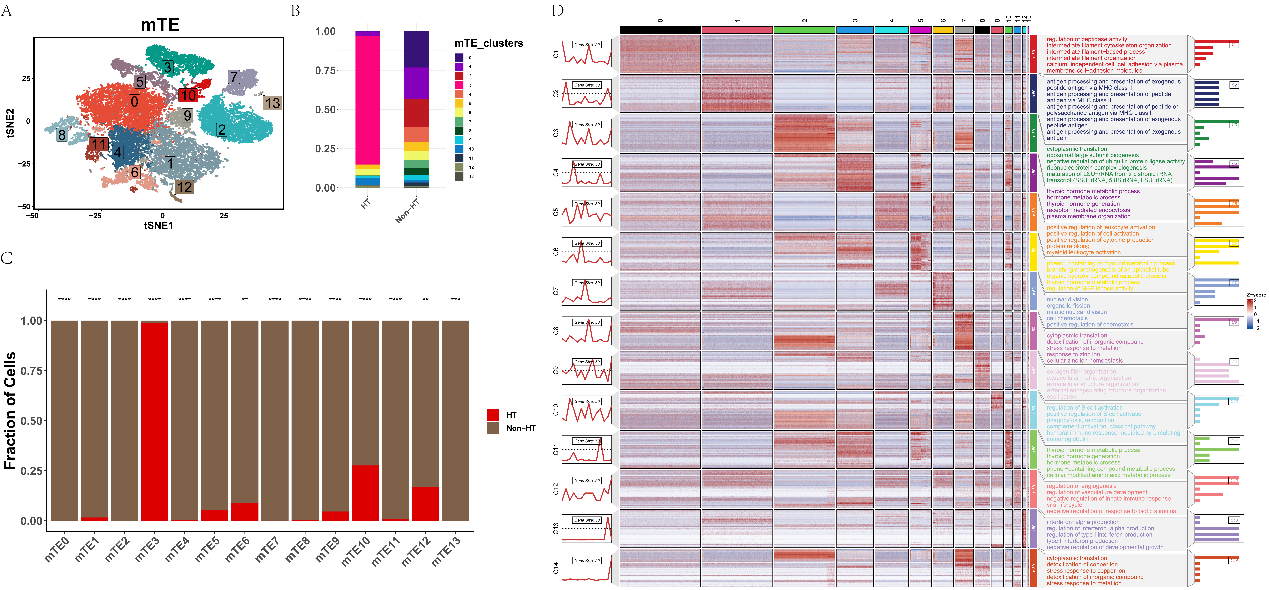


Fig. S1 scRNA-seq analysis of malignant thyroid epithelial cells (mTE) (A) t-SNE plot showing mTE colored by clusters (n = 14). (B) Horizontal bar charts showing the relative abundance of various clusters between HT and Non-HT. (C) Post-hoc analysis of each cluster between HT and Non-HT,*P<0.05, **P < 0.01, ***P < 0.001, ****P<0.0001. (D) GO enrichment analysis of top 50 marker genes in 14 clusters.


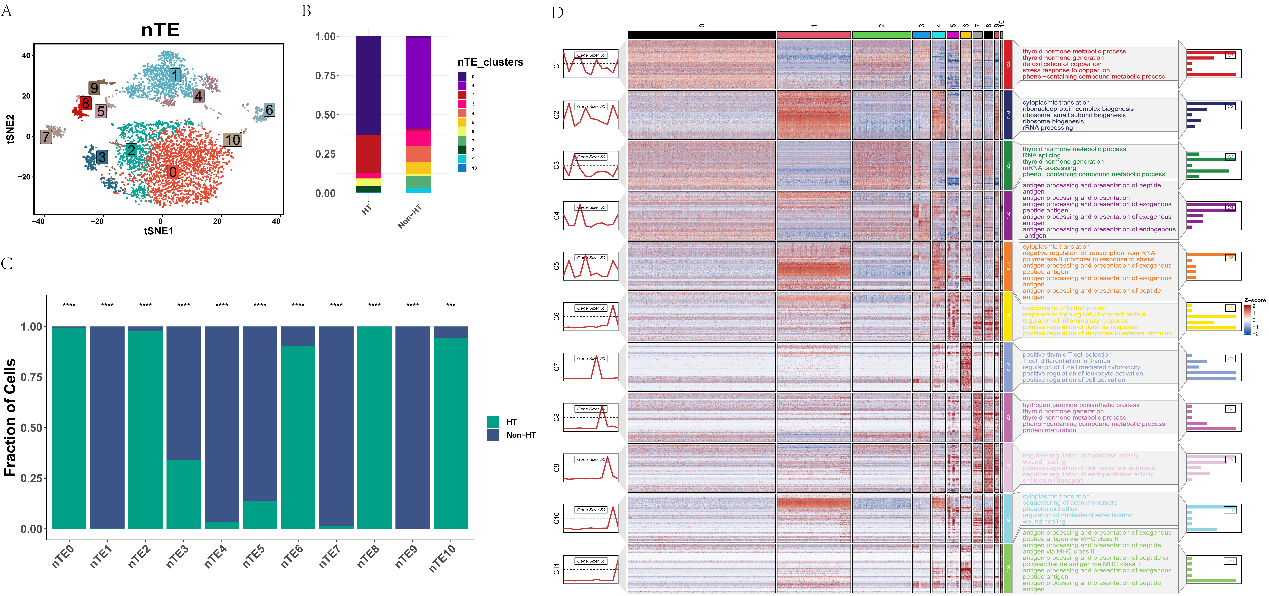


Fig. S2 scRNA-seq analysis of non-malignant thyroid epithelial cells (nTE) (A) t-SNE plot showing nTE colored by clusters (n = 11). (B) Horizontal bar charts showing the relative abundance of various clusters between HT and Non-HT. (C) Post-hoc analysis of each cluster between HT and Non-HT,*P<0.05, **P < 0.01, ***P < 0.001, ****P<0.0001. (D) GO enrichment analysis of top 50 marker genes in 11 clusters.


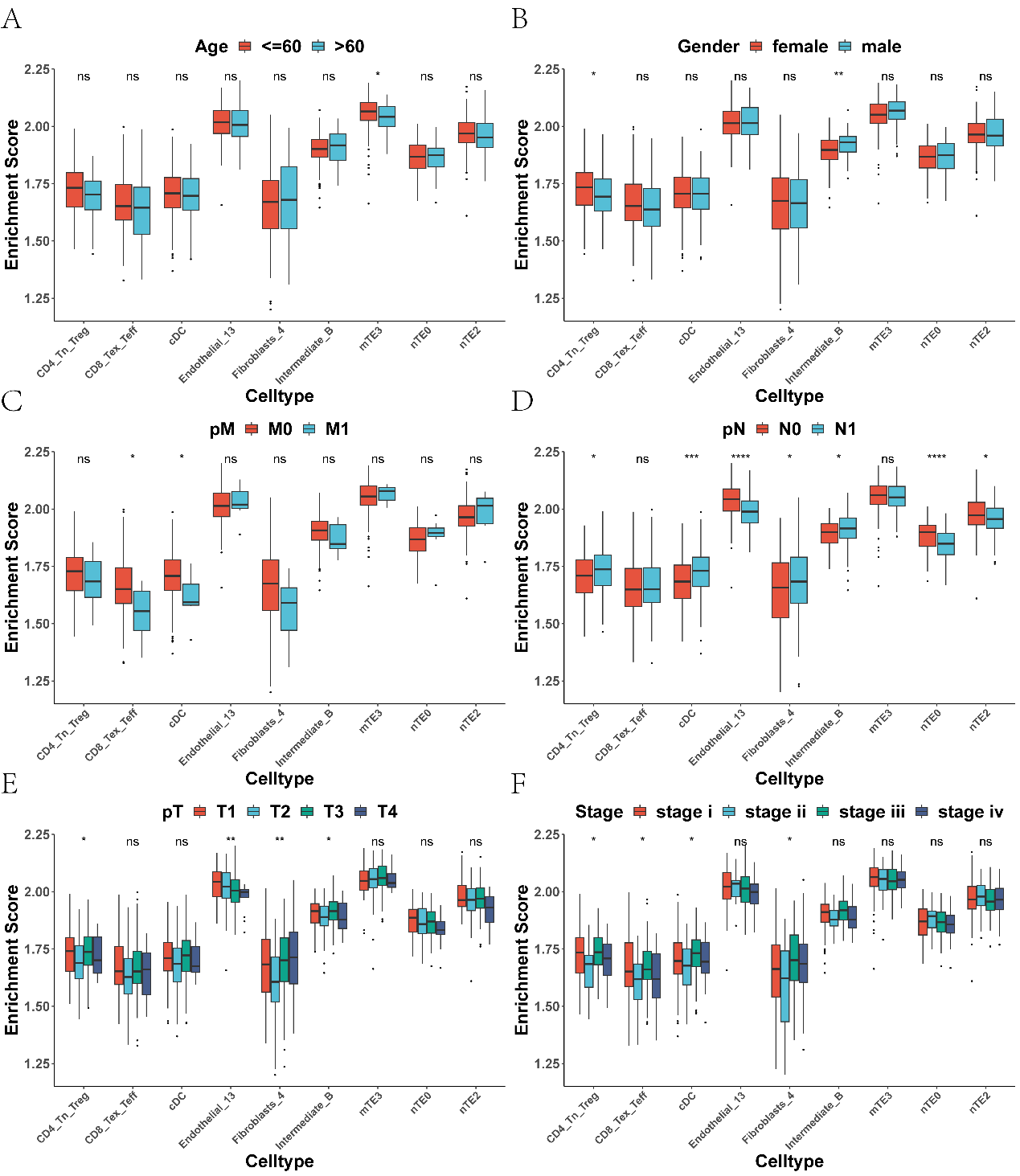

Fig. S3 Relationship between HASC content and clinical features. (A-F) Box plot of the HASC content in different ages (A), gender(B), pM(C), pN(D), pT(E), stage(F). *P < 0.05; **P < 0.01; ***P < 0.001; ****P < 0.0001.
